# Annexin A4-nuclear factor-κB feedback circuit regulates cell malignant behavior and tumor growth in gallbladder cancer

**DOI:** 10.1038/srep31056

**Published:** 2016-08-05

**Authors:** Hou-Shan Yao, Chang Sun, Xin-Xing Li, Yi Wang, Kai-Zhou Jin, Xiao-Ping Zhang, Zhi-Qian Hu

**Affiliations:** 1 Department of General Surgery, Shanghai Chang Zheng Hospital, Second Military Medical University, Shanghai 200003, China; 2 Department of General Surgery, Jinling Hospital, Medical School of Nanjing University, Nanjing 210002, Jiangsu Province, China; 3 Institute of Intervention Vessel, Tongji University, North Zhongshan Road, Shanghai 200070, China; 4 Department of Nuclear Medicine, Shanghai Tenth People’s Hospital, Tongji University, Shanghai 200072, China

## Abstract

Gallbladder cancer (GBC) is the most common malignant tumor of the biliary system. However, the mechanisms underlying its tumor initiation, progression, and metastasis are not yet fully understood. The annexin A4 (*ANXA4*) gene is highly expressed in GBC tissues and may play an important role in the initiation and progression of this disease. In this study, we examined the up-regulation of *ANXA4* in human GBC tissues and cell lines. Elevated ANXA4 correlated well with invasion depth in GBC patients and predicted a poor prognosis. *In vitro*, GBC-SD and NOZ cells with *ANXA4* knockdown demonstrated increased apoptosis and inhibited cell growth, migration, and invasion. Interactions between ANXA4 and nuclear factor-κB (NF-κB) p65 proteins were detected. *In vivo*, *ANXA4* knockdown inhibited tumor growth of GBC cells in nude mice and down-regulated the expression of downstream factors in the NF-κB signaling pathway. Taken together, these data indicate that up-regulation of *ANXA4* leads to activation of the NF-κB pathway and its target genes in a feedback regulatory mechanism via the p65 subunit, resulting in tumor growth in GBC.

Gallbladder cancer (GBC) is the most common malignancy of the biliary tract and exhibits a remarkable geographic variability, with particularly high incidences in Latin America, Korea, and Japan^1^. The survival of patients with GBC is typically only about 6 months after diagnosis^2^. Screening markers, early diagnosis, and fast, effective treatment are thus essential for controlling this disease. Although a model for gallbladder carcinogenesis has been proposed^3^, the underlying molecular mechanisms are still not fully understood. However, accumulating evidence implicates the involvement of a number of genes, including those encoding cyclooxygenase-2 (*COX-2*)^4^, epidermal growth factor receptor (*EGFR*)^3^, fragile histidine triad (*FHIT*)^5^, inducible nitric oxide synthase (*iNOS*)^6^, mucus 5AC (*MUC5AC*)^7^, the p16/cyclin D1/CDK4 pathway^8^, *p53*^9^, a member of the nuclear factor (NF)-κB DNA transcription complex, and vascular endothelial growth factor (*VEGF*)^10^.

We previously identified annexin A4 (*ANXA4*) as a potential biomarker for GBC^11^. This protein is markedly up-regulated in the cytoplasm of primary GBC tissues compared with normal tissues, and has also been implicated in other adenocarcinomas^12^, including gastric cancer^13^, lung cancer^14^, mesothelioma^15^, colorectal cancer^16^ and ovarian cancer^17^. Duncan *et al.* reported that ANXA4 expression was significantly increased in colorectal cancer compared with normal colon, and that upregulation of ANXA4 was associated with advanced tumor stage and decreased survival^16^.

ANXA4 is a member of the annexin family, which includes proteins that aggregate on cell membranes and bind phospholipids in a calcium-dependent manner. Although the detailed physiological roles of ANXA4 are unclear, previous studies have reported that it has important functions in membrane permeability, exocytosis, and the regulation of chloride conductance^18^,^19^,^20^. With regard to cancer, elevated expressions of ANXA4 has been reported in various clinical epithelial tumors^13^,^14^,^15^,^16^,^17^, in which ANXA4 regulates downstream signals, such as hyaluronan mediated motility receptor, lysosomal-associated membrane protein 2, Akt, cyclin-dependent kinase 1 and p21 in a Ca^2+^-assisted manner^13^, thus stimulating the NF-κB pathway to suppress apoptosis and ultimately inducing tumor invasiveness and metastasis^16^,^21^. Furthermore, ANXA4 has also shown to be involved in tumor dissemination and anti-cancer drug resistance^22^. These previous studies suggested that ANXA4 triggers a signaling cascade leading to increased epithelial cell proliferation, ultimately promoting carcinogenesis.

In this study, we examined the potential role of *ANXA4* in the early tumorigenic mechanism of GBC by examining its expression and knockdown in GBC tumors and cell lines, and its involvement in the NF-κB pathway.

## Results

### ANXA4 is highly expressed in GBC tumors and cell lines

**Figure 1:**
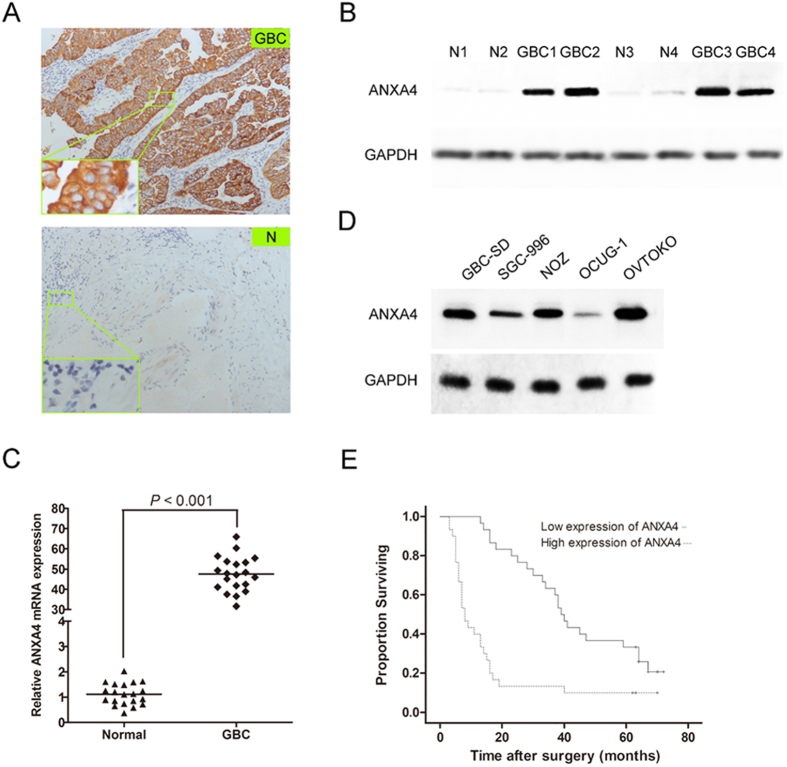
Expression of ANXA4 in human gallbladder cancer tissues and cell lines. **(A)** Representative photographs of immunohistochemical staining of ANXA4 protein (brown) in gallbladder cancer (GBC) tumor and normal tumor-adjacent tissues (N). Original magnification (×100, full; ×400, partial enlargement). **(B)** Western blot analysis of ANXA4 protein accumulation in gallbladder cancer and normal adjacent tissues. ANXA4 protein levels were significantly elevated in GBC tissues compared with normal adjacent tissues. GAPDH as a positive control. **(C)** *ANXA4* mRNA expression levels in gallbladder cancer tissues (n = 20) were significantly higher than in normal adjacent specimens (n = 20, *P* < 0.001), according to quantitative RT-PCR. **(D)** Western blot analysis of ANXA4 and GAPDH protein levels in GBC-SD, SGC-996, NOZ and OCUG-1 gallbladder cancer cell lines. GBC-SD and NOZ cells expressed the highest levels of ANXA4 protein. Human ovarian clear cell adenocarcinoma OVTOKO cells as a positive control. **(E)** OS of gallbladder cancer patients associated with ANXA4 expression. Kaplan–Meier analysis of the GBC patients, indicated the poorer survival of patients with high ANXA4 expression (χ^2^ value = 19.371, *P* < 0.001). The median OS of GBC patients with low ANXA4 expression was 39 (95% confidence interval (CI), 34.7–43.3) months, while the median OS of high-ANXA4-expressing patients was 8 (95% CI, 5.7–10.3) months. The actual 3-year and 5-year OS rates for patients with low ANXA4 expression (63.3% and 33.3%, respectively) were higher than those for patients with high ANXA4 expression (13.3% and 10.0%, respectively, *P* < 0.001).

Immunohistochemical staining revealed that ANXA4 protein was abundantly present in human GBC tumors, compared with weak or no expression in normal tumor-adjacent tissues (Fig. 1A). High ANXA4 expression was noted in 11/20 (55%) GBC tissue samples and 0/20 adjacent matched noncancerous tissue samples (*P* < 0.001). Consistent with the immunohistochemical data, western blot analysis showed that ANXA4 protein levels were significantly elevated in GBC tumors compared with normal adjacent tissues (Fig. 1B). Real-time polymerase chain reaction (RT-PCR) analysis indicated that *ANXA4* mRNA was expressed at significantly higher levels in all gallbladder tumor specimens compared with normal tissues (Fig. 1C). Western blot analysis of four human GBC cell lines (GBC-SD, SGC-996, NOZ, and OCUG-1) indicated that GBC-SD and NOZ cells expressed the highest levels of ANXA4 protein (Fig. 1D).

### ANXA4 overexpression correlated with invasion depth and lymph node metastasis and predicted poor prognosis in GBC patients

We further investigated the clinical significance of differential ANXA4 expression in GBC tissues by analyzing expression levels in a larger patient population. High expression of ANXA4 was detected in 30/60 (50.0%) GBC samples. As shown in Table 1, high expression of ANXA4 was positively associated with lymph node metastasis (*P* < 0.001), invasion depth (*P* = 0.028), and TNM stage (*P* = 0.010). Kaplan–Meier survival analysis showed that patients with high ANXA4 expression had poorer overall survival (OS) than patients with low ANXA4 expression (*P* < 0.001) (Fig. 1E). The median OS of GBC patients with low ANXA4 expression was 39 months (95% confidence interval (CI), 34.7–43.3) compared with 8 months (95% CI, 5.7–10.3) in patients with high ANXA4 expression. The actual 3-year and 5-year OS rats of patients with low ANXA4 expression were 63.3% and 33.3%, respectively, compared with only 13.3% and 10.0%, respectively, in patients with high ANXA4 expression. Multivariate Cox proportional hazards analysis revealed that ANXA4 expression was an independent prognostic marker for OS in GBC patients (*P* < 0.001), and that invasion depth (*P* < 0.001) and lymph node metastasis (*P* = 0.003) were also significantly associated with OS.

### *ANXA4* knockdown inhibited gallbladder cell growth and increased apoptosis

**Figure 2:**
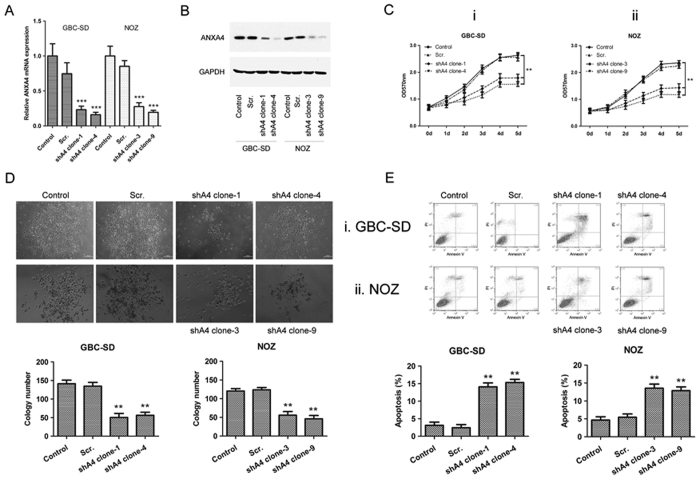
*ANXA4* knockdown affected gallbladder cancer cell growth and apoptosis *in vitro*. Knockdown of endogenous *ANXA4* expression was confirmed by RT-PCR **(A)** and western blot **(B)**. GAPDH as an internal control. Expression levels of *ANXA4* mRNA and protein were obviously down-regulated in *ANXA4*-knockdown clones (shA4 clone number). Scr., scrambled control, expressing non-targeting control shRNA. Non-transfected cells as control. The effects of shRNA-mediated knockdown of *ANXA4* in GBC-SD and NOZ cells on malignant behavior were examined using several methods. Data are presented as mean ± SD compared with the control. **(C)** Cell proliferation was assessed by methylthiazol tetrazolium assay. *ANXA4*-knockdown cells proliferated at significantly more slowly than non-transfected cells (**P < 0.01). **(D)** Colony formation assays. Colony formation in GBC-SD and NOZ cells was significantly inhibited after transfection with *ANXA4* shRNA (**P < 0.01). **(E)** Cell apoptosis assessed by flow cytometry. *ANXA4* shRNA increased apoptotic rates of GBC-SD and NOZ cells (**P < 0.01).

**Figure 3:**
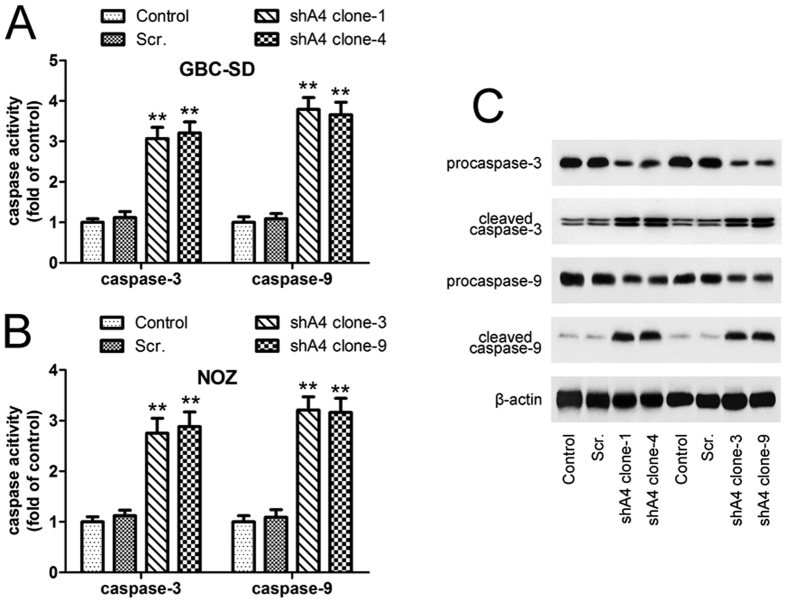
Effect of *ANXA4* knockdown on caspase activation in GBC-SD and NOZ cells. Caspase-3 and -9 activities were determined by caspase activity assay in GBC-SD (**A**) and NOZ (**B**) cells. Caspase-3 and -9 activities were significantly increased in *ANXA4*-knockdown cells. Data are presented as mean ± SD from three independent experiments. **P < 0.01 compared with control. (**C**) Pprotein expression levels of total caspase-3 and -9 were decreased in *ANXA4*-knockdown clones, while their cleaved forms increased, as shown by western blot analysis.

The high *ANXA4*-expressing GBC cell lines, GBC-SD and NOZ, were transfected with lentivirus-based short hairpin RNA (shRNA) targeting human *ANXA4* (shA4) or the corresponding scrambled construct, with non-transfected cells as a control. To rule out clonal effects, *in vitro* and *in vivo* experiments were performed in two *ANXA4* knockdown clones. RT-PCR and western blot analyses revealed that *ANXA4* mRNA (Fig. 2A) and protein (Fig. 2B) levels were reduced, while the expression levels of other annexins, *ANXA1*, *ANXA2* and *ANXA3* (Supplementary Figure S1), remained unchanged in the *ANXA4*-knockdown clones. *ANXA4*-knockdown cells proliferated (Fig. 2C) and formed colonies (Fig. 2D) at significantly slower rates than non-transfected cells. Flow cytometric analysis revealed significantly higher levels of annexin V binding in *ANXA4* shRNA-transfected cells compared with control cells, indicating increased apoptosis (Fig. 2E). We determined which caspase molecules were involved in *ANXA4* shRNA-induced apoptosis by measuring caspase-3 and -9– activities in *ANXA4* shRNA-transfected cells by caspase activity assays. Compared with untreated control cells, *ANXA4* shRNA significantly induced caspase-3 and -9 activities in GBC-SD and NOZ cells (Fig. 3A,B). Western blot analysis showed that full-length procaspase-3 and procaspase-9 were significantly decreased in *ANXA4*-knockdown cells, while their cleaved forms were increased (Fig. 3C).

### *ANXA4* knockdown inhibited GBC cell migration and invasion

**Figure 4:**
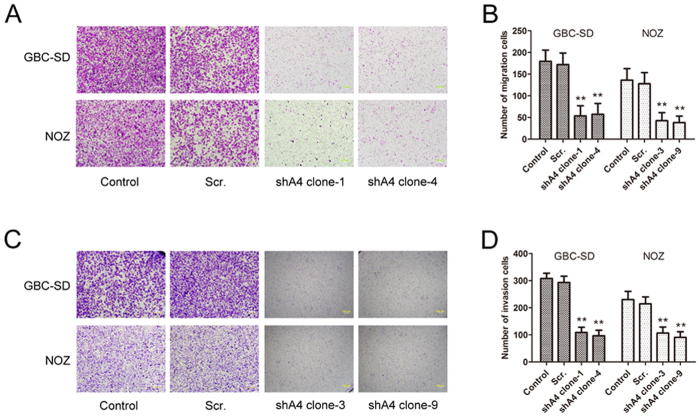
*ANXA4* knockdown affected gallbladder cancer cell migration and invasion *in vitro*. **(A)** Cell migration and **(C)** cell invasion were assessed by Transwell assays. **(B)** Migrating and **(D)** invading cells that invaded the bottom well were counted in 10 visual fields. Data are presented as mean ± SD from three independent experiments. Compared with the control groups, *ANXA4*-knockdown significantly inhibited the migration and invasion abilities of GBC-SD and NOZ cells (**P < 0.01).

We determined the influence of *ANXA4* down-regulation on the migration and invasion abilities of GBC-SD and NOZ cells using the Transwell system. Compared with control cells, cell migration (Fig. 4A,B) and invasion (Fig. 4C,D) were significantly reduced in *ANXA4*-knockdown cells.

### p65 subunit is required for feedback regulation between ANXA4 and NF-κB

**Figure 5:**
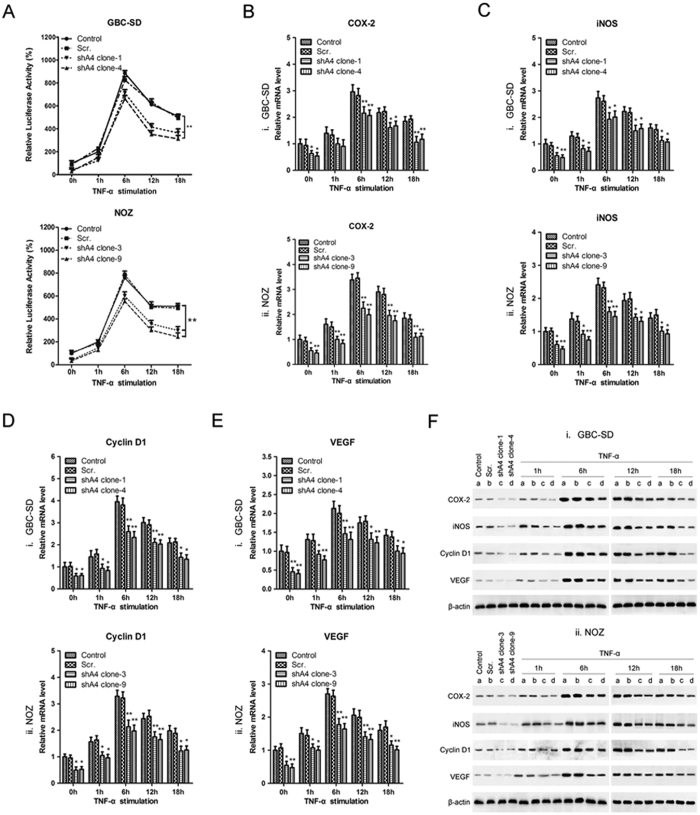
Knockdown of *ANXA4* modulated NF-κB transcriptional activity and expression of its downstream target genes. **(A)** NF-κB transcriptional activity was assayed in *ANXA4*-knockdown clones of (i) GBC-SD and (ii) NOZ after TNF-α stimulation using a reporter assay system. NF-κB transcriptional activity in *ANXA4* shRNA cells was significantly decreased compared with the control. The expression levels of NF-κB downstream targets COX-2, iNOS, cyclin D1, and VEGF were determined by RT-PCR **(B–E)** and western blot analyses **(F)** in *ANXA4*-knockdown (i) GBC-SD and (ii) NOZ cells after TNF-α stimulation. *ANXA4* shRNA significantly reduced the expression of these factors at the mRNA and protein levels. β-actin expression was used as a control. All data are presented as mean ± SD from three independent experiments. *P < 0.05, **P < 0.01, compared with control. *ANXA4* shRNA-transfected cell clones: shA4 clone-1, c; shA clone-4, d; scrambled control (b, Scr.); non-transfected cells (a, Control).

We explored the effects of *ANXA4* knockdown on transcriptional activation of NF-κB in GBC cells by luciferase assays in GBC-SD and NOZ cells transfected with *ANXA4* shRNA or scrambled constructs, with non-transfected cells as a control. As shown in Fig. 5A, *ANXA4*-knockdown significantly reduced NF-κB transcriptional activity both before and after tumor necrosis factor (TNF)-α stimulation, compared with controls. *ANXA4*-knockdown significantly reduced mRNA levels of the NF-κB downstream target genes *COX-2, iNOS, cyclin D1* and *VEGF* before and after TNF-α stimulation (Fig. 5B–E). Western blot analysis indicated that the corresponding protein levels were also notably reduced in *ANXA4*-knockdown clones (Fig. 5F).

**Figure 6:**
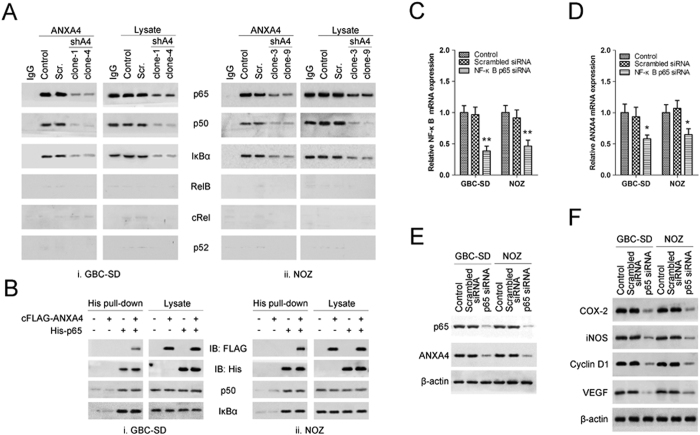
ANXA4 interacted with the p65 subunit of NF-κB. Interactions between the ANXA4 and p65 proteins were assessed in (i) GBC-SD and (ii) NOZ cells. **(A)** Proteins were extracted from *ANXA4* knockdown clones, scrambled control (Scr.) and non-transfected cells (Control) and subjected to western blot analysis with antibodies specific for the indicated proteins, or β-actin as a control. Right panels: untreated cell lysates; left panels: immunoprecipitates (IP) produced with antibodies specific for ANXA4 or IgG as a control. **(B)** Nuclear extracts from GBC-SD or NOZ cells transfected with vectors containing cFLAG-tagged ANXA4 and/or His-tagged p65 were subjected to western blot analysis with His-, FLAG-, p50-, IκBα- or control β-actin antibodies. Right panels: untreated extract; left panels: pellets from His-tag pull-down analysis. Cells transfected with vectors containing NF-κB *p65* siRNA, the corresponding scrambled siRNA, or non-transfected controls were subjected to RT-PCR **(C)** or western blot analysis **(D)** using primers for *p65* and *ANXA4* mRNA, or antibodies specific for p65 or ANXA4 proteins, respectively. NF-κB *p65* siRNA down-regulated *p65* and *ANXA4* mRNA expression. Data are presented as mean ± SD from three independent experiments. *P < 0.05, **P < 0.01 compared with control. **(E)** Cells transfected with vectors containing NF-κB *p65* siRNA, the corresponding scrambled siRNA, or non-transfected controls were subjected to western blot analysis using antibodies specific for COX-2, iNOS, cyclin D1, and VEGF or control β-actin antibodies. NF-κB *p65* siRNA reduced the protein expression levels of the NF-κB downstream targets.

We investigated the interaction between ANXA4 and NF-κB subunits by co-immunoprecipitation of endogenous proteins in extracts from *ANXA4*-knockdown cells, scrambled control cells, and non-transfected control cells. Immunoprecipitation of endogenous ANXA4 revealed a strong association with endogenous p65 in GBC-SD and NOZ cells (Fig. 6A), and this interaction was reduced in *ANXA4*-knockdown cells. We also detected p50 and inhibitor of κB (IκBα) in this immunoprecipitate, but not the other subunits RelB, cRel and p52 (Fig. 6A). The interaction of p65 with ANXA4 was confirmed by His pull-down assays (Fig. 6B). His pull-down experiments also showed no direct interaction of ANXA4 with p50 or IκBα, suggesting that the associations of p50 and IκBα with ANXA4 were indirect and occurred through an inhibitory p50-p65-IκBα complex (Fig. 6B).

We examined any effects of feedback regulation of *p65* on *ANXA4* expression using NF-κB *p65* small interfering RNA (siRNA) knockdown GBC-SD and NOZ cells. The presence of NF-κB *p65* siRNA reduced p65 and ANXA4 mRNA (Fig. 6C,D) and protein (Fig. 6E) expression levels. Moreover, the expression levels of the NF-κB downstream targets COX-2, iNOS, cyclin D1, and VEGF proteins were down-regulated in *p65*-knockdown cells. Taken together, these results suggest that the p65 subunit is required for feedback regulation between ANXA4 and NF-κB.

### *ANXA4* knockdown inhibited tumor growth of GBC cells in nude mice by inhibiting the NF-κB signaling pathway

**Figure 7:**
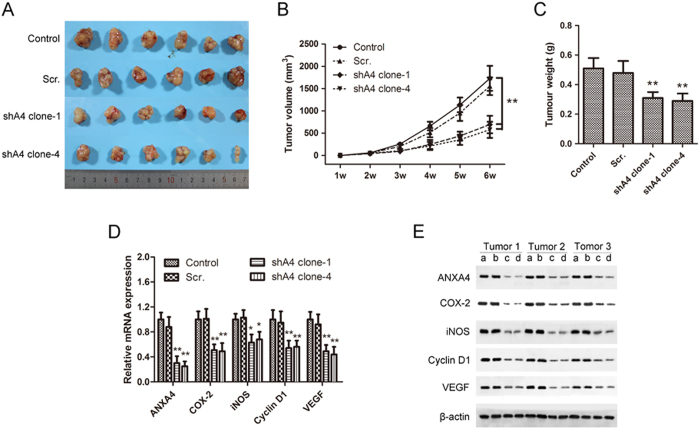
*ANXA4* knockdown inhibited tumor growth of gallbladder cancer cells in nude mice and down-regulated the expression of key downstream targets of the NF-κB signaling pathway. *ANXA4* knockdown (c, shA4 clone-1; d, shA clone-4), scrambled control (b, Scr.), or non-transfected control (a) GBC-SD cells were injected subcutaneously into six nude mice each. After 6 weeks, mice were sacrificed and the tumors were removed. All excised tumors are shown in **(A)**. **(B)** Tumor volume was measured and calculated every week. Tumor volume was significantly decreased in mice injected with *ANXA4*-knockdown cells compared with the control group (**P < 0.01). **(C)** Tumor weight was significantly reduced in mice injected with *ANXA4*-knockdown cells (**P < 0.01 compared to the control group). The mRNA and protein expression levels of ANXA4 and NF-κB signaling pathway factors COX-2, iNOS, cyclin D1, and VEGF in tumors were determined by **(D)** RT-PCR and **(E)** western blot, respectively. mRNA and protein expression levels were significantly decreased in tumor tissues from mice injected with ANXA4 shRNA-transfected cells. Data are presented as mean ± SD; *P < 0.05, **P < 0.01 compared with blank control levels.

Nude mice were injected with *ANXA4*-knockdown, scrambled control, and non-transfected control cells to induce tumors, and the tumors were harvested every week for 6 weeks (Fig. 7A). Tumor volume and weight were significantly decreased in mice injected with *ANXA4*-knockdown cells (Fig. 7B,C). These tumors also exhibited lower accumulations of ANXA4, COX-2, iNOS, cyclin D1, and VEGF mRNAs (Fig. 7D) and proteins (Fig. 7E) compared with tumors from mice injected with control cells.

## Discussion

The dismal prognosis of GBC has been attributed to typical delays in diagnosis, the difficulty of radical resections (currently the only potentially curative therapy), and insufficient therapies for advanced-stage patients. In addition, few predictable biomarkers are available for GBC prognosis in the clinical environment. A recent analysis comparing the complete exomes from 57 gall-bladder tumors with normal tissues aimed at identifying the somatic mutation spectrum reported a high rate of C > T/G > A somatic mutation in GBC tissue^23^. This study identified several GBC driver mutations and indicated ErbB signaling as a core pathway, thus providing potentially important information for the development of targeted therapies. We previously provided the first report identifying ANXA4 as a potential biomarker for GBC using proteomic analysis^11^. In the current study, we showed that overexpression of ANXA4 correlated well with invasion depth as well as lymph node metastasis, and predicted poor survival in GBC patients. *ANXA4* knockdown in GBC cells inhibited cell proliferation and increased apoptosis compared with control GBC cells. These results are consistent with the reported role of *ANXA4* in both of these processes. *ANXA4* knockdown also inhibited cell migration and invasion, in accordance with the reported involvement of this gene in pathogenic proliferation and invasion of adenocarcinoma cells^12^.

ANXA4 has been implicated in chemoresistance in ovarian cancer^12^, lung cancer^24^ and malignant mesothelioma^15^. The mechanism of ANXA4-induced platinum resistance may be mediated in part by increased efflux of cellular platinum via the copper transporter ATP7A^25^,^26^, while modulation of Ca^2+^-dependent NF-κB transcriptional activity may also play a role. After etoposide treatment, ANXA4 co-translocated to the nucleus with the p50 subunit, subsequently enhancing the transcriptional activity of NF-κB and ultimately inducing resistance to etoposide-associated apoptosis^27^. Morimoto *et al.* recently proposed a novel mechanism for ANXA4-induced chemoresistance whereby calcium-binding sites in the annexin repeats of ANXA4 were responsible for resistance to platinum-based drugs by elevating the intracellular chloride concentration^28^. The role of ANXA4 in cancer and chemoresistance suggests that it could represent a potential therapeutic target for cancer treatment.

ANXA4 has been shown to interact with members of the mammalian NF-κB signaling pathway, including p105 and p50^27^. Jeon *et al.* revealed that ANXA4 interacted with the NF-κB p50 subunit and modulated the transcriptional activity of NF-κB in a calcium-dependent manner. In the presence of high Ca^2+^ levels, ANXA4 co-translocated to the nucleus with the p50 subunit, consequently increasing the transcriptional activity of NF-κB, and ultimately inducing resistance to etoposide-associated apoptosis. The NF-κB family includes five proteins forming a protein complex that controls DNA transcription, and has been linked to a number of cancers^29^. Activation of NF-κB p65 (also known as v-rel avian reticuloendotheliosis viral oncogene homolog A) is associated with several types of cancer and is believed to play a major role in tumorigenesis^30^. Lui and Brown showed that phosphorylation of p65 in follicular thyroid carcinomas correlated with translocation to the nucleus and gene expression of COX-2, interleukin-8 and glutathione S-transferase-π^31^. In addition, Rho-kinase mediates TNF-α-stimulated nuclear translocation of NF-κB p65 and subsequent DNA binding activity, and this mediation may be an important step in macrophage proliferation^32^. We conclude that p65 can interact with some annexins, and these interactions play an important role in carcinogenesis.

Receptor interacting protein (RIP)-1 has been reported to be up-regulated in GBC, promoting NF-kB (p65) activation^33^. Ubiquitination of RIP1 plays an important role in TNF-α-induced IKK/NF-κB activation^34^,^35^. The expression of many genes encoding transcription factors and cell signaling modulators are also altered during GBC pathogenesis, suggesting that the interactions with their corresponding partners are also affected. However, in terms of NF-κB activation, the association between RIP1 upregulation and ANXA4 upregulation in GBC is unknown. The interaction mechanisms between *ANXA4* and other genes, including RIP1 will be an interesting goal for future studies aimed at extending our understanding of GBC pathogenesis.

The results of this study showed that the p65 subunit was required for the feedback circuit between ANXA4 and NF-κB, which has been implicated in tumor development and progress. To the best of our knowledge, this is the first report of a feedback circuit between ANXA4 and NF-κB. The existence of this feedback circuit suggests that ANXA4 interacts with the p65 subunit in such a way that loss of ANXA4 decreases the transcriptional activity of NF-κB, and inhibition of NF-κB subsequently suppresses ANXA4 expression. This hypothesis is strengthened by the fact that deregulation and knockdown of *ANXA4* in GBC cells corresponded to changes in expression levels of NF-κB downstream target genes, such as COX-2, iNOS, cyclin D1, and VEGF. These NF-κB downstream target genes were down-regulated in tumors from mice injected with *ANXA4*-knockdown clones, indicating that NF-κB plays an important role in tumor growth regulated by *ANXA4* in GBC. In addition, NF-κB *p65* knockdown significantly reduced p65 and ANXA4 mRNA and protein expression. Similarly, Campbell *et al.* reported that ANXA6 interacted with p65, and that ANXA6 overexpression resulted in increased NF-κB activity, increased catabolic events in articular chondrocytes, and accelerated cartilage destruction^36^. However, the mechanism whereby NF-kB regulates ANXA4 expression is unknown.

To confirm the feedback regulatory mechanism inhibiting tumor growth *in vivo*, we injected GBC cells into nude mice to induce tumors. *ANXA4*-knockdown GBC cells produced smaller tumors than GBC cell lines expressing high levels of *ANXA4*, associated with lower accumulation of ANXA4 and NF-κB, including COX-2, iNOS, cyclin D1, and VEGF. Taken together, these observations suggest that ANXA4 interacts with the NF-κB p65 subunit and modulates the transcriptional activity of the NF-κB pathway in an ANXA4–NF-κB feedback circuit. ANXA4 has been implicated in several cancers, and the current results suggest that therapy targeting ANXA4 may suppress the NF-κB signaling pathway, further reversing *ANXA4* overexpression in a feedback regulatory mechanism and resulting in inhibition of tumor growth. We postulate that overexpression of *ANXA4* during GBC development and progress activates the NF-κB signaling pathway, which in turn drives ANXA4 expression, resulting in carcinogenesis and invasiveness. *ANXA4* knockdown thus provides promising prospects for developing GBC therapies based on the ANXA4–NF-κB feedback circuit in gallbladder cells.

## Materials and Methods

### Patients and tissues

Tissue specimens, including 60 GBC tumor specimens and 20 normal tumor-adjacent tissues, were obtained from 60 primary GBC patients who underwent surgery at Shanghai Chang Zheng Hospital affiliated with the Second Military Medical University, None of the patients had received any preoperative antineoplastic therapy. The study was conducted in accordance with the human subject guidelines approved by the Scientific and Ethical Committee of the Second Military Medical University, Shanghai, China. All samples were obtained by experienced surgeons and examined by experienced pathologists from 2006 to 2008. Informed consent was obtained from all patients or their relatives for the use of tissues in experimental procedures. Formalin-fixed, paraffin-embedded samples were sectioned at 4-μm thicknesses, and immunohistochemical staining was performed as described below. In addition, 20 self-paired GBC and normal tumor-adjacent tissue specimens were snap frozen in liquid nitrogen and stored at −80 °C following surgery for quantitative real-time RT-PCR analysis.

Clinical staging was performed according to the American Joint Committee on Cancer, 6th Edition guidelines^37^. No distant metastasis was detected in any of the patients, and all patients achieved clear resection margins (R0), defined as complete removal of the neoplasm without macroscopic residual tumor and with negative histologic margins. Stage I GBC patients underwent cholecystectomy; stage II patients underwent radical surgeries including cholecystectomy, regional lymphadenectomy, and resection of the gallbladder fossa; stage III patients underwent radical tumor resection combined with regional lymphadenectomy and wedge resection of the liver. Information on relevant patient clinicopathological variables including age, sex, tumor size, histology, differentiation, depth of tumor and lymph node metastasis was collected, and follow-up information was also recorded. OS was calculated from the day of surgery to death or January 2013. All patients were followed-up until death or January 2013. After surgery, patients with high-risk stage III GBC were treated with chemotherapy using gemcitabine.

### Immunohistochemical staining and assessment

Immunohistochemical staining was performed using the standard immunoperoxidase procedure^38^. Briefly, serial 4 μm sections were cut from formalin-fixed and paraffin-embedded tissue, dewaxed in xylene, rehydrated in alcohols, and then incubated with fresh 3% hydrogen peroxide for 20 min at room temperature. After washing with phosphate-buffered saline (PBS), the tissue sections were antigen-retrieved by heating in a microwave for 13 min in a citric acid buffer solution (pH 6.0). Sections were blocked with appropriate normal serum in PBS. The specific antibodies were incubated with the sections overnight in humidified boxes at 4 °C. The sections were then washed with PBS for 5 min followed by incubation with an UltraSensitive S-P Kit (Maixin-Bio, Fuzhou, China), according to the manufacturer’s instructions. After exposure to stable 3,3-diaminobenzidine for 5–10 min, slides were counterstained with hematoxylin, dehydrated, and mounted. Control sections were incubated with PBS instead of the primary antibody. Microscopic images were captured with a BX41 light microscope (Olympus Optical Co., Tokyo, Japan) equipped with an Olympus DP70 digital camera and image analysis software (analySIS 3.2, Soft Imaging System, Hanover, Germany). Three sections were randomly selected for each group, and five high-power (×400) fields were observed randomly for each section.

Immunohistochemical reactions were analyzed using a previously described semiquantitative scoring system, in which the final immunoreaction score was expressed as the product of the intensity and quantity scores^39^. The percentage of positive cells was graded as follows: 0, negative; 1, <10% positive cells; 2, 11–50% positive cells; 3, 51–80% positive cells; 4, >80% positive cells. The staining intensity was graded as follows: 0, negative; 1, weakly positive; 2, moderately positive; and 3, strongly positive. The product of the above two scores was defined as the protein expression level, and was given a numerical value from 0–12 for further analysis. High ANXA4 expression in the tumor was defined by a score ≥ 6, and low expression as a score < 6. All staining results were independently evaluated by three different pathologists who were blinded to patient outcomes.

### Cell lines

Human GBC cell lines were obtained as follows: GBC-SD and SGC-996, from the Shanghai Institutes for Biological Sciences (Shanghai, China), and OCUG-1 and NOZ were from the Japanese Collection of Research Bioresources JCRB cell Bank (Osaka, Japan). Human ovarian clear cell adenocarcinoma cell lines OVTOKO were also obtained from the JCRB Cell Bank. GBC-SD and OVTOKO cells were cultured in RPMI 1640 medium (Gibco, NY, USA), SGC-996 and OCUG-1 cells were cultured in Dulbecco’s Modified Eagle’s Medium (Gibco), and NOZ cells were cultured in William’s E medium (Gibco), with all media supplemented with 10% fetal bovine serum (Gibco), 10 units/ml penicillin, and 10 mg/ml streptomycin (1% P/S, Thermo Scientific HyClone, UT, USA). All cell lines were incubated at 37 °C in a humidified atmosphere containing 5% CO_2_ and subcultured during the logarithmic phase. GBC cell lines and OVTOKO cells were authenticated by short tandem repeat profiling, as described previously. The short tandem repeat profiles are presented in Supplementary Figure S2.

### Reagents and plasmids

C-terminal FLAG-tagged *ANXA4* expression plasmids (cFLAG-*ANXA4*) were generated by PCR and subcloned into pcDNA3.1/Zeo (Thermo Fisher Scientific, MA, USA). His-tagged *p65* expression plasmids (His-*p65*) were generated by PCR and subcloned into pcDNA4/HisMax (Thermo Fisher Scientific). Knockdown of NF-κB *p65* was performed using SignalSilence^®^ NF-κB *p65* siRNA I (Cell Signaling Technology, MA, USA).

The following antibodies were used in this study: goat anti-ANXA4, mouse anti-NF-κB p65, p50, c-Rel, RelB, cRel and p52, goat anti-COX-2, mouse anti-iNOS, rabbit anti-VEGF, and mouse anti-glyceraldehyde 3-phosphate dehydrogenase (GAPDH; Santa Cruz Biotechnology, Inc., CA, USA), as well as rabbit anti-cyclin D1, rabbit anti-6X His tag, mouse anti-β-actin (Abcam, Cambridge, England), and mouse anti-FLAG (Sigma, MO, USA).

### Lentiviral shRNA knockdown of *ANXA4*

*ANXA4* knockdown in GBC-SD and NOZ cells was accomplished by stably expressing lentivirus-based shRNA targeting human *ANXA4*, in a manner similar to the method described in previous studies^40^. Briefly, the pLKO.1 vector (Open Biosystems, AL, USA) was employed, and *ANXA4* shRNA sequence (sense: 5′-CCG GGC ACA CTT CAA GAG ACT CTT CGG ATC CGA AGA GTC TCT TGA AGT GTG CTT TTT G-3′; antisense: 5′-AAT TCA AAA AGC ACA CTT CAA GAG ACT CTT CGG ATC CGA AGA GTC TCT TGA AGT GTG C-3′) was subcloned into the pLKO.1 vector. A non-targeting control shRNA (scrambled control, Scr.) was obtained from Sigma-Aldrich (MO, USA). Recombinant lentivirus was produced by cotransfection with three helper vectors, pRSV-REV, pMDLg/pRRE and pMD2G, and a target vector pLKO.1-puro-shRNA into HEK-293T cells. GBC-SD and NOZ cells were then infected with recombinant lentivirus for 24-h, and stably expressing cells were selected with puromycin. The efficiency of knockdown was determined by quantitative real-time PCR and western blot analysis.

### Tumor xenograft animal model

Balb/c athymic nude mice (24 males, 6 weeks old, 18–21 g) were provided by the Shanghai Laboratory Animal Center (Chinese Academy of Science, China) and housed in specific pathogen-free conditions. All animal experimental procedures were approved by the Experimental Animal Center of the Second Military Medical University, and the methods were carried out in accordance with the approved guidelines. Tumor xenograft assay of GBC-SD cells *in vivo* was performed as described previously^41^,^42^. Mice were randomly assigned to four groups (six mice per group): shA4 clone-1, shA4 clone-4, scrambled control and GBC-SD (control). Lentivirus-transfected cells were administered by subcutaneous injection (0.2 ml PBS containing 5 × 10^6^ GBC-SD cells/ml). After development of a palpable tumor, the tumor volume (V) was determined by measuring the two perpendicular dimensions with calipers every week and calculated using the formula: V (mm^3^) =1/6πab^2^, where a was the larger and b the smaller dimension of the tumor. Six weeks after inoculation, the mice were sacrificed and tumors were resected and weighed. A portion of each tumor was selected for RT-PCR and western blot analysis. All measures were performed independently by two different technicians who were blinded to the group allocation during the experiment.

### Other methods

Quantitative real-time RT-PCR, western blot, cell proliferation assay, colony formation assay, cell migration and invasion assays, flow cytometric analysis of apoptosis, luciferase reporter assay, co-immunoprecipitation, and His pull-down assay were performed using standard protocols. See Supplementary Methods for more details.

### Statistical analysis

Results are expressed as mean ± standard deviation (SD) based on a minimum of three replicates. Differences between groups were evaluated using SPSS version 19.0 statistical software (IBM Corporation, NY, USA). Results were compared between two groups using Student’s *t*-tests (two-tailed), and among more than two groups by one-way analysis of variance. The relationships between *ANXA4* expression levels and various clinicopathologic characteristics were analyzed by non-parametric Mann-Whitney tests. Survival rates were determined using the Kaplan–Meier method. *P* < 0.05 was considered statistically significant.

## Additional Information

**How to cite this article**: Yao, H.-S. *et al.* Annexin A4-nuclear factor-κB feedback circuit regulates cell malignant behavior and tumor growth in gallbladder cancer. *Sci. Rep.* **6**, 31056; doi: 10.1038/srep31056 (2016).

## Supplementary Material

Supplementary Information

## Figures and Tables

**Table 1:** Relationship between ANXA4 expression and clinical pathological characteristics in patients with primary gallbladder cancer.

Variablen (n = 60)	ANXA4		*P* value
	high expression	low expression	
Age (years)			
<60	12	10	0.592
≥60	18	20	
Sex			
Male	10	11	0.787
Female	20	19	
Size (cm)			
<3	10	12	0.592
≥3	20	18	
Histology			
Adenocarcinoma	27	28	0.640
Others	3	2	
Differentiation			
G1/G2	23	20	0.390
G3	7	10	
Depth of tumor			
T1 + T2	3	10	0.028
T3	27	20	
Lymph node metastasis			
Yes	22	6	<0.001
No	8	24	
TNM			
I + II	2	10	0.010
III	28	20	
