# Supplementary material for: Annexin A4-nuclear factor-κB feedback circuit regulates cell malignant behavior and tumor growth in gallbladder cancer
Source: Sci Rep. 2016 Aug 5;6:31056. doi: 10.1038/srep31056 (PMC4974512; doi:10.1038/srep31056)
Supplement: Supplementary Information [file srep31056-s1.doc]

**Annexin A4-nuclear factor-κB feedback circuit regulates cell malignant behavior and tumor growth in gallbladder cancer**

Running title: *ANXA4* -NF-κB feedback circuit regulates tumor growth in GBC

Hou-Shan Yao^1, *^, Chang Sun^2, *^, Xin-Xing Li^1^, Yi Wang^1^, Kai-Zhou Jin^1^, Xiao-Ping Zhang^3, 4, #^, Zhi-Qian Hu^1, #^

^1^ Department of General Surgery, Shanghai Chang Zheng Hospital, Second Military Medical University, Shanghai 200003, China

^2^ Department of General Surgery, Jinling Hospital, Medical School of Nanjing University, Nanjing 210002, Jiangsu Province, China

^3^ Institute of Intervention Vessel, Tongji University, North Zhongshan Road, Shanghai 200070, China

^4^ Department of Nuclear Medicine, Shanghai Tenth People’s Hospital, Tongji University, Shanghai 200072, China

^*^ These authors contributed equally to this paper.

^#^ Corresponding authors:

Zhi-Qian Hu, MM, Department of General Surgery, Shanghai Chang Zheng Hospital, Second Military Medical University, 415 Feng Yang Road, Shanghai 200003, China; Email: huzq62@163.com; Tel.: 86-21-81885591; Fax: 86-21-81885591

Xiao-Ping Zhang, PhD, Institute of Intervention Vessel, Tongji University, No. 727 North Zhongshan Road, Shanghai 200070, China and Department of Nuclear Medicine, Shanghai Tenth People’s Hospital, Tongji University, No. 301, Yanchang Road, Shanghai 200072, China; Email: zxpsibs@163.com; Tel.: 86-21-64373365; Fax: 86-21-64373365

**Supplementary Methods**

**RNA extraction and quantitative RT-PCR**

The mRNA levels of *ANXA1*, *ANXA2*, *ANXA3*, *ANXA4*, *p65*, *COX-2*, *iNOS*, *Cyclin D1* and *VEGF* were quantified by RT-PCR. After total RNA extraction using TRIzol Reagent (Thermo Fisher Scientific), cDNA was synthesized using a RevertAid™ First Strand cDNA Synthesis Kit (Fermentas, MA, USA). RT-PCR was performed with a SYBR Green RT-PCR Kit (Qiagen, Hilden, Germany) using the primers listed in Supplementary Table S1, on an ABI Prism 7300 RT-PCR detection system (Applied Biosystems, Foster City, CA, USA). The reaction conditions were as follows: 95°C for 10 min followed by 40 cycles of 95°C for 15 s, 60°C for 1 min, and 72°C for 30 s. The relative expression levels of each gene were calculated and normalized using the 2^−ΔΔCt^ method relative to detected *GAPDH* or β-actin levels. All reactions were repeated in triplicate.

**Western blot analysis**

Proteins from tissue samples and cultured cells were determined by western blot analysis as described previously.[^1^](#_ENREF_1) Cells were lysed and the supernatant was collected by centrifugation at 8000 × *g* for 10 min at 4°C. Protein concentrations were determined using a BCA protein quantitation kit (Thermo Fisher Scientific), according to the manufacturer’s instructions. Equivalent amounts of proteins were then separated by 12% sodium dodecyl sulfate-polyacrylamide gel electrophoresis (SDS-PAGE) and transferred to a polyvinylidene difluoride membrane (Millipore, Bedford, MA, USA). The membranes were subsequently immunoblotted with the appropriate primary antibody at 4°C overnight, and then further incubated with the corresponding horseradish peroxidase-conjugated secondary antibody. The target proteins were visualized using an enhanced chemiluminescent detection system (Pierce, Rockford, IL, USA), imaged on the Bio-Rad chemiluminescence imager. Equal protein loading was confirmed by the expression of GAPDH or β-actin.

**Cell proliferation assay**

Cell proliferation ability was assessed by methylthiazol tetrazolium (MTT) assay. Briefly, GBC-SD and NOZ cells were incubated in 96-well plates to a density of 8 × 10^3^ cells per well. Ten microliters of sterile 5 mg/ml 3-(4,5-dimethylthiazol-2-yl)-2,5-diphenyltetrazolium bromide (Sigma) was added to each well and incubated for 4 h at 37°C. Subsequently, 100 µl of dimethyl sulfoxide (Sigma) was added and the samples were mixed thoroughly for 10 min. The optical density at 570 nm was obtained with a Fluostar Optima spectrophotometer (BMG Labtech GmbH, Ortenberg, Germany). Cell proliferation was determined at days 1, 2, 3, 4, and 5. Each growth assay was repeated in triplicate.

**Colony-formation assay**

Colony-formation assays were performed using 200 cells plated into 35 mm culture plates for 10 days. The resulting cell clones were counted using a BX45-72P15 inverted microscope (Olympus, Tokyo, Japan). A cell clone was scored as positive when the number of cells in the clone exceeded 50. The experiment was repeated four times.

**Analysis of apoptosis by flow cytometry**

Approximately 1 × 10^5^ cells were collected, washed with cold phosphate-buffered saline (PBS), centrifuged, resuspended in 100 µl binding buffer containing 2.5 µl fluorescein isothiocyanate-conjugated annexin V and 1 µl of 100 µg/ml propidium iodide and incubated for 15 min at room temperature in the dark. At least 10,000 events were collected and analyzed by flow cytometry (Becton, Dickinson and Company, CA, USA). The percentage of apoptotic cells was reported.

**Caspase activity assay**

Caspase activities were measured using Caspase-Glo 3, 9 activity assay kits (Promega, Madison, WI, USA) according to the manufacturer’s recommendations. Data were collected using a Wallac Victor 3 microplate reader (Perkin Elmer, Waltham, MA, USA) at 405 nm.

**Cell migration and invasion assays**

Cell migration and invasion were assayed as described previously [^2^](#_ENREF_2) using Transwell permeable supports with 8 μm pore size (Costar, MA, USA). Cells were suspended in serum-free medium and seeded into Transwell inserts, either uncoated (for migration assay) or coated (for invasion assay) with growth factor-reduced Matrigel (BD Biosciences, MA, USA). The bottom wells were filled with complete medium, and cells were fixed with methanol and stained with a crystal violet solution after 24 h. The number of cells that penetrated the membrane was determined by counting the mean cell number in five randomly selected high-power fields.

**Luciferase reporter assay**

For NF-κB luciferase assay, cells were co-transfected with a *Photinus pyralis* firefly NF-κB-specific luciferase reporter vector (pGL4.32 [luc2P/NF-κB-RE/Hygro], Promega, WI, USA) and a control pRL-TK *Renilla* luciferase vector (Promega). Luciferase activity was assessed using the Dual-Luciferase Assay Reporter System (Promega). *P. pyralis* luciferase activity was normalized to *R.* *reniformis* luciferase activity to account for the transfection efficiency in each well. The values represent an average of three experiments, each performed in triplicate.

**Co-immunoprecipitation**

Cultured GBC-SD and NOZ cells were extracted with buffer containing 50 mM Tris·HCl, pH 7.4, 1% Nonidet P-40, 0.1% Triton X-100, 150 mM NaCl, 5 mM EDTA, and proteinase-inhibitor mixture. A portion of the lysate was subjected to western blot analysis. The remaining lysate was subjected to co-immunoprecipitation using Dynabeads Protein G (Thermo Fisher Scientific), according to the manufacturer’s instructions. Briefly, the Dynabeads were incubated with antibodies for ANXA4 or IgG for 10 min with agitation, followed by incubation with 1 mg protein from cell extracts for 30 min at room temperature with agitation. After washing, the antibody–antigen complex was eluted, prepared and analyzed on NuPage Novex Bis-Tris Gels (Life Technologies Corporation, CA, USA), according to the manufacturer's instructions. Proteins in the gels were transferred to nitrocellulose filters. After blocking with a low-fat milk protein solution, blotted proteins were immunostained first with primary antibodies and then with peroxidase-conjugated secondary antibodies. The signal was detected using the enhanced chemiluminescence detection system (Pierce) as described previously [^3^](#_ENREF_3).

**Pull-down of His-tagged proteins**

Pull-down of His-tagged proteins was performed as described previously.[^4^](#_ENREF_4) Briefly, cFLAG-*ANXA4* and His-p65 constructs were cotransfected into GBC-SD and NOZ cells. Cells were lysed in Ni-NTA lysis buffer (20 mM NaH_2_PO_4_, 300 mM NaCl, 5 mM imidazole, and 0.05% Tween 20) and incubated at 4°C for 30 min. After centrifugation at 13,000 rpm for 30 min, cell lysates containing cFLAG-ANXA4 and His-p65 were mixed with Ni-NTA-agarose beads (Qiagen, CA, USA) at 4°C for 6 h with agitation. Nonspecifically bound proteins were removed by washing with wash buffer and bound proteins were eluted with 1 × SDS-PAGE sampling buffer containing 250 mM imidazole, and were subsequently separated by 10% SDS-PAGE followed by immunoblotting with anti-FLAG and anti-His antibodies.

**References**

1 Zhang, J. T. *et al.* Norcantharidin inhibits tumor growth and vasculogenic mimicry of human gallbladder carcinomas by suppression of the PI3-K/MMPs/Ln-5gamma2 signaling pathway. *BMC Cancer* **14**, 193, (2014).

2 Tao, J. *et al.* Down-regulation of FoxM1 inhibits viability and invasion of gallbladder carcinoma cells, partially dependent on inducement of cellular senescence. *World J Gastroenterol* **20**, 9497-9505, (2014).

3 Campbell, K. A. *et al.* Annexin A6 interacts with p65 and stimulates NF-kappaB activity and catabolic events in articular chondrocytes. *Arthritis Rheum* **65**, 3120-3129, (2013).

4 Jeon, Y. J. *et al.* Annexin A4 interacts with the NF-kappaB p50 subunit and modulates NF-kappaB transcriptional activity in a Ca2+-dependent manner. *Cell Mol Life Sci* **67**, 2271-2281, (2010).

**Supplementary Table S1. Primers used in this study.**

| **Gene** | **Forward (5′–3′)** | **Reverse (5′–3′)** |
| --- | --- | --- |
| *ANXA1* | TGCTTTCTCTTGCTAAGGGTG | CTGGTGGTAAGGATGGTATTG |
| *ANXA2* | ACACCTGCTCAGTATGACGCTTC | CATCCTCTGCTCTTCTACCCTTT |
| *ANXA3* | AACATCTGGTGACTTCCG | AATTTGTCTTCATCCGTG |
| *ANXA4* | TAAAACGCCTACAGCTGCCT | TAAGCTTTGAAATGCAAGTACAGC |
| *p65* | CAACCCCTTCCAAGTTCCTAT | CACCTCAATGTCCTCTTTCTG |
| *COX-2* | TTGGGTGTCAAAGGTAAAAA | AACTGATGCGTGAAGTGCTG |
| *iNOS* | TCCTGTCCCCTTTCTACTAC | CCTCTGATTTTCCTGTCTCT |
| *Cyclin D1* | CTGGAGCCCGTGAAAAAGAG | AGCGTGTGAGGCGGTAGTAG |
| *VEGF* | TCGTTGCGAGTGTGTCTGT | ATTCTTTCGGCTGTGGGGC |
| *GAPDH* | ACCACAGTCCATGCCATCAC | TCCACCACCCTGTTGCTGTA |
| *β-actin* | GGCATCCACGAAACTACCTT | TCCTGCTTGCTGATCCACAT |

**Supplementary figures**


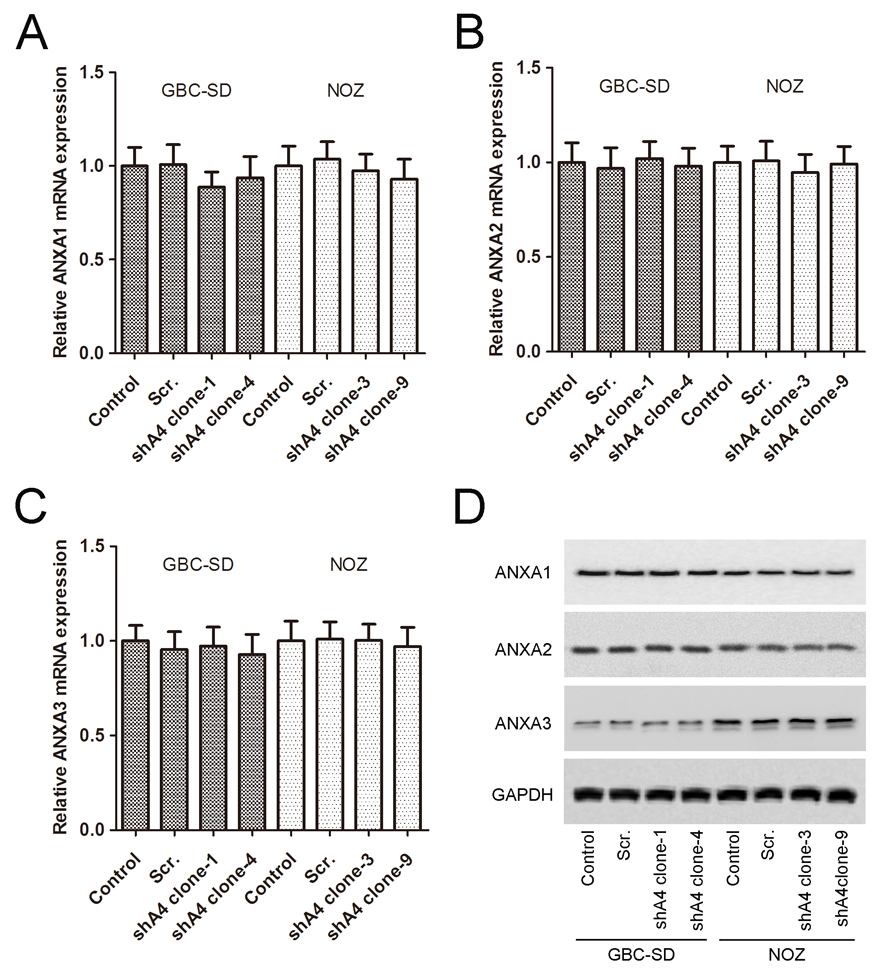


**Supplementary Figure S1. Effect of *ANXA4* knockdown on expression of other annexins members**

mRNA expression levels of other annexins *ANXA1* (A), *ANXA2* (B) and *ANXA3* (C) remained unchanged in *ANXA4*-knockdown clones, as shown by quantitative RT-PCR (*P* > 0.05). (D) Protein expression levels of these annexins were also unchanged, according to western blot analysis.


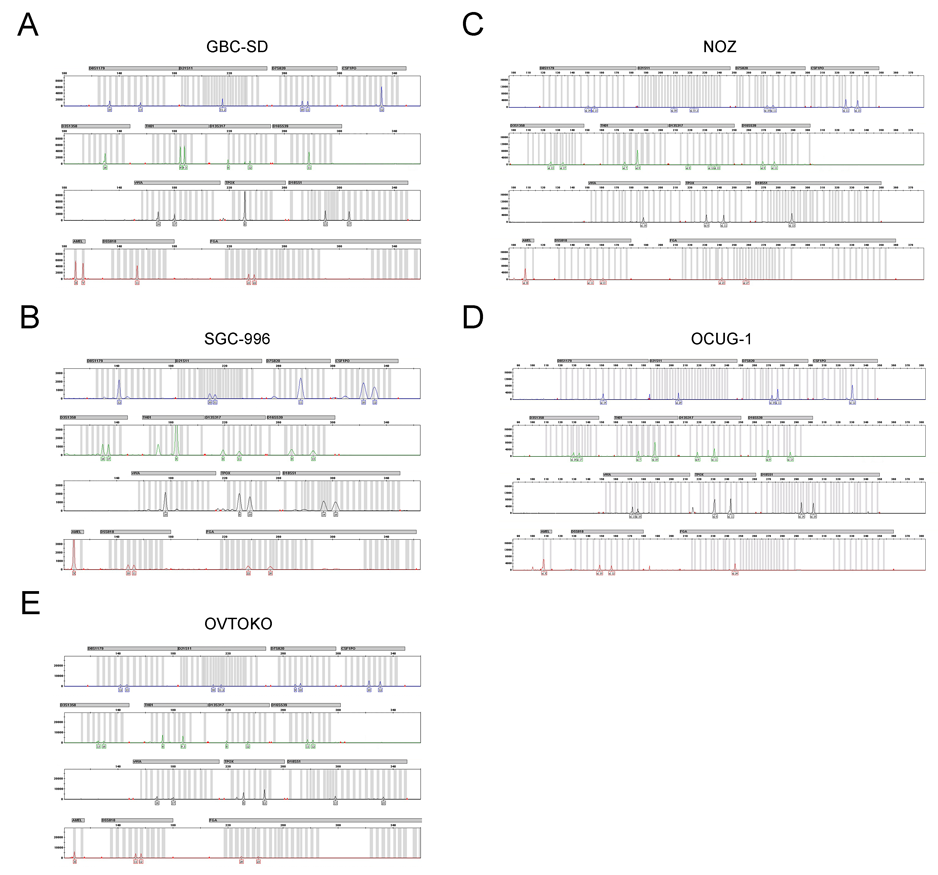


**Supplementary Figure S2. Short tandem repeat profiles of GBC and OVTOKO cell lines.**

We identified four unique human gallbladder cancer cell lines: (A) GBC-SD, (B) SGC-996, (C) NOZ, and (D) OCUG-1, and one unique human ovarian clear cell adenocarcinoma cell line OVTOKO (E). DNA from these cell lines was analyzed and blue, green, black and red amplified short tandem repeat peaks and allele classifications were shown. The number(s) below each peak represent the number of repeats at that locus. The loci are as follows: blue (left to right): D8S1179, D21S11, D7S820, CSF1PO; green: D3S1358, TH01, D13S317, D16S539; black: vWA, TPOX, D18S51; and red: AMEL (amelogenin), D5S818, FGA.
